# Filling in the gaps: estimating numbers of chlamydia tests and diagnoses by age group and sex before and during the implementation of the English National Screening Programme, 2000 to 2012

**DOI:** 10.2807/1560-7917.ES.2017.22.5.30453

**Published:** 2017-02-02

**Authors:** Nastassya L Chandra, Kate Soldan, Ciara Dangerfield, Bersabeh Sile, Stephen Duffell, Alireza Talebi, Yoon H Choi, Gwenda Hughes, Sarah C Woodhall

**Affiliations:** 1 National Infection Service, Public Health England, London, United Kingdom

**Keywords:** Chlamydia, surveillance, sexually transmitted infections, mathematical modelling, screening, national chlamydia screening programme

## Abstract

To inform mathematical modelling of the impact of chlamydia screening in England since 2000, a complete picture of chlamydia testing is needed. Monitoring and surveillance systems evolved between 2000 and 2012. Since 2012, data on publicly funded chlamydia tests and diagnoses have been collected nationally. However, gaps exist for earlier years. We collated available data on chlamydia testing and diagnosis rates among 15–44-year-olds by sex and age group for 2000–2012. Where data were unavailable, we applied data- and evidence-based assumptions to construct plausible minimum and maximum estimates and set bounds on uncertainty. There was a large range between estimates in years when datasets were less comprehensive (2000–2008); smaller ranges were seen hereafter. In 15–19-year-old women in 2000, the estimated diagnosis rate ranged between 891 and 2,489 diagnoses per 100,000 persons. Testing and diagnosis rates increased between 2000 and 2012 in women and men across all age groups using minimum or maximum estimates, with greatest increases seen among 15–24-year-olds. Our dataset can be used to parameterise and validate mathematical models and serve as a reference dataset to which trends in chlamydia-related complications can be compared. Our analysis highlights the complexities of combining monitoring and surveillance datasets.

## Introduction

Genital infection with *Chlamydia trachomatis* (‘chlamydia’) is the most commonly diagnosed sexually transmitted infection (STI) in Europe [1,2]. If left untreated, genital chlamydia infection can cause serious complications in both women and men, including pelvic inflammatory disease (PID), ectopic pregnancy, tubal factor infertility and epididymitis [3]. Prevalence is generally highest in young adults [4-6]. A recent systematic review found estimates of prevalence in Europe and other high-income countries among sexually-experienced ≤ 26-year-olds ranged from 3.0% to 5.3% in women and 2.4% to 7.3% in men [7].

Chlamydia control activities vary across Europe, ranging from case management for diagnosed cases to opportunistic testing among asymptomatic individuals [8]. In England, in recognition of the public health importance of genital chlamydia infection, the National Chlamydia Screening Programme (NCSP) was introduced in 2003 and was active nationwide by 2008. The programme aims to reduce chlamydial infections and associated complications by detecting and treating asymptomatic infections through opportunistic screening. The NCSP recommends all sexually active under-25-year-olds be tested for genital chlamydia infection annually or on change of sexual partner (whichever is the most frequent) and those who test positive should be offered a re-test around 3 months after treatment [3]. Screening is accessible through a range of providers, which include general practitioners (GPs), pharmacies, contraception services, sexual health and reproductive services and pregnancy termination services [9]. In 2013, over 1.7 million chlamydia tests were carried out among 15–24-year-olds in both specialist sexual health services (genitourinary medicine clinics (GUM)) and non-specialist services with over 139,000 positive chlamydia results reported (hereafter ‘diagnoses’ refers to ‘positive chlamydia results’) [10].

Understanding changes in chlamydia tests and diagnosis will better inform us of the NCSP’s effects on chlamydia infections and can facilitate programme evaluation in regards to both genital chlamydia prevalence and understanding related complications. Gaining an understanding of the NCSP’s impact is also useful for other European countries considering the optimal approach to chlamydia control [11]. Annual data on testing and diagnoses are required for parameterisation of mathematical models which explore the effect of chlamydia screening on chlamydia prevalence. As acknowledged by authors of previous mathematical modelling studies of chlamydia screening, one of the limitations of current models is the availability of robust data on chlamydia tests and diagnoses in this context. A comprehensive overview of testing practices before and during NCSP implementation can also facilitate interpretation of trends in chlamydia-related complications. However, since 2000, national monitoring and surveillance systems that include reported chlamydia tests and diagnoses in England have evolved considerably [10,12,13] resulting in reporting gaps in the data. These gaps include non-reported data from specific years, settings or age groups; missing age and sex data; and referrals between settings. In this paper, we estimate chlamydia testing and diagnosis rates by age group among men and women aged 15 to 44 years between 2000 and 2012 using data from several datasets and a sample of GP records, combined with data-driven and evidence-based assumptions, to fill the gaps in these data. Our estimates of chlamydia testing and diagnosis rates are needed to provide robust data for mathematical models that can be used to evaluate chlamydia control activities.

## Methods

### Data sources

**Figure 1:**
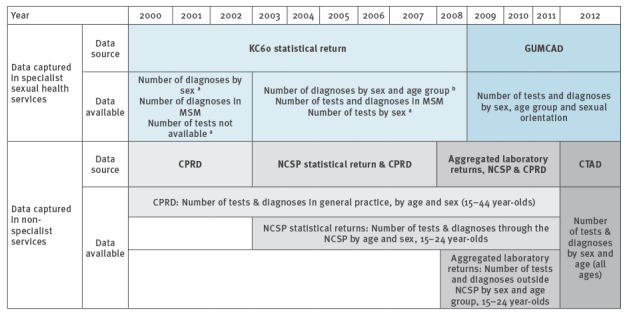
Schematic of available chlamydia activity data from national monitoring and surveillance systems in specialist sexual health services and non-specialist services, England, 2000–2012

CPRD:  Clinical Practice Research Datalink; CTAD:  Chlamydia Testing Activity Dataset; GUMCAD: genitourinary medicine clinic activity dataset; MSM: men who have sex with men; NCSP: National Chlamydia Screening Programme.

Data on chlamydia tests and diagnoses were compiled from several data sources. The data available varied according to the years and test settings covered and the extent to which data were provided by age group (Figure 1).

GUMCAD is a collection of electronic patient-level data that captures tests and diagnoses provided in commissioned sexual health services. CTAD is a collection of electronic patient-level data that captures all publicly funded chlamydia testing activity and diagnoses in England.

^a^ Includes uncomplicated and complicated chlamydia captured separately from each other in the KC60 statistical return.

^b^ Includes uncomplicated and complicated chlamydia captured separately from each other in the KC60 statistical return but complicated chlamydia is not broken down by age group.

### Specialist sexual health services

Numbers of chlamydia tests and diagnoses carried out in specialist sexual health services were derived from the KC60 statistical return (2000–2008) and genitourinary medicine clinic activity dataset (GUMCAD, 2009–2012). Details of these datasets are reported elsewhere [12]. Briefly, the KC60 was a mandatory statistical return which provided an aggregated dataset of diagnoses and services delivered in specialist sexual health services in England up to 2008. Between 2000 and 2002 the number of tests were not recorded; diagnoses were broken down by sex but not age. Tests were included from 2003 and broken down by sex but not age; diagnoses were available by sex and age for uncomplicated chlamydial infections but not available by age for complicated chlamydial infections. GUMCAD, which was introduced in 2009, is a mandatory disaggregated data return of STI diagnoses and services provided submitted by all specialist services across England [12]. The number of tests and diagnoses are available by sex, age and sexual orientation.

### Non-specialist sexual health services

Chlamydia tests and diagnoses made outside specialist sexual health services were derived from three nationally collated datasets:

NCSP statistical returns (2003–2011): a disaggregated return from testing venues of all chlamydia tests and diagnoses among 15–24-year-olds tested as part of the NCSP between 2003 and September 2012.
Aggregated laboratory return (2008–2011): this return captured data from all laboratories that collected tests and diagnoses among 15–24-year-olds reported from outside of specialist sexual health services and not as part of the NCSP between April 2008 and September 2012 (e.g. in hospitals or in GP settings not carried out as part of the NCSP).
Chlamydia Testing Activity Dataset (CTAD, 2012): CTAD is a disaggregated data return from laboratories that replaced the NCSP statistical return and aggregated laboratory return in 2012. CTAD captures all publicly funded chlamydia tests and diagnoses in England for all ages [10,13].

Before the introduction of CTAD in 2012, national monitoring and surveillance systems in England did not cover chlamydia testing carried out among those aged 25 years and over attending non-specialist clinics [12,13]. We therefore supplemented the datasets described above using data from the clinical practice research datalink (CPRD). CPRD comprises anonymised patient-level medical records of registered patients in a sample (ca 10%) of GPs across the United Kingdom (UK) [14-16]. Attendances for chlamydia tests and diagnoses among men and women aged 15–44 years between 2000 and 2011 were identified using a pre-defined selection of ‘Read Codes’ (diagnostic codes used in primary care, data not shown). Duplicate codes within a 42-day period were considered part of the same episode and subsequently excluded.

With the exception of data from CPRD, estimates of testing coverage (number of tests as a percentage of the population) and diagnosis rates (number of diagnoses per 100,000 population) were calculated using population denominators provided from the Office of National Statistics [17]. Testing and diagnosis rates reported in CPRD were calculated by dividing the number of tests and diagnoses by the person-years contributed by the registered practice population in each year by sex and age group [14].

### Data and evidence base to address the known limitations of national monitoring and surveillance systems

**Figure 2:**
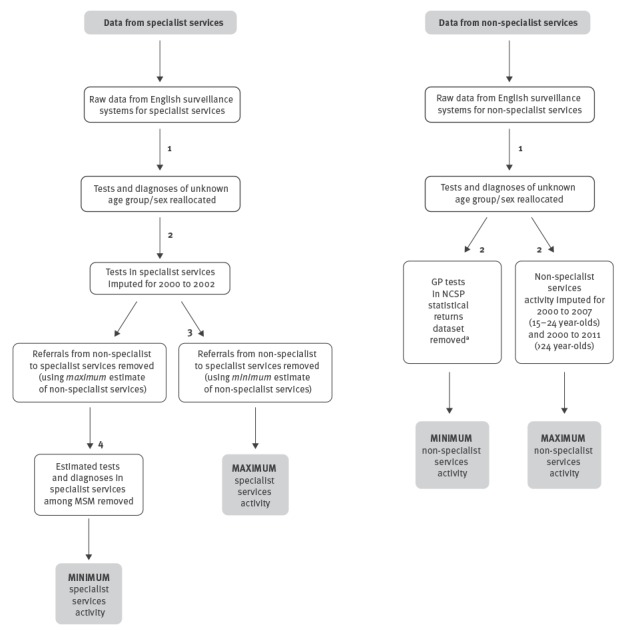
Flowchart summarising combinations and adjustments to the data from specialist sexual health services and non-specialist sexual health services to construct plausible minimum and maximum estimates of chlamydia tests and diagnosis rates by sex and age group, England, 2000–2012

GP: general practice; MSM: men who have sex with men; NCSP: National Chlamydia Screening Programme.

After combining the datasets, we made a series of adjustments to resolve gaps in the data and calculate minimum and maximum plausible estimates of chlamydia testing coverage and diagnosis rates for each age group by sex and year in both specialist and non-specialist services (Figure 2). We define adjustments to the data as modifications (as described below) rather than statistical adjustments. The datasets were adjusted to account for missing age and sex variables, differences in case definitions (complicated and uncomplicated chlamydial infections), referrals between non-specialist and specialist services, and missing data for certain years. Some adjustments to the data could be undertaken using more than one possible method. To justify the methods used, sensitivity analyses, statistical tests and comparisons to other research were used (Table 1).

Adjustment number 1:  dealing with data of unknown age group or sex; Adjustment number 2: imputing non-reported data from a specific year, setting or age group; Adjustment number 3: allowing for referrals from non-specialist to specialist settings; Adjustment number 4: removal of MSM from the minimum-activity estimate for specialist settings.

^a^ It is unknown whether chlamydia tests carried out in GP settings, but as part of the NCSP, would also be captured within GP records. Therefore, where there was potential duplication (2003–2007), GP tests were removed from the NCSP statistical returns dataset.

**Table 1:** Rationale for methods used for adjustments to the data for estimating numbers of chlamydia tests and diagnoses by age group and sex, before and during the implementation of the National Screening Programme, England, 2000–2012.

Adjustment and adjustment number^a^	Assumption	Data and evidence base for assumption
Reallocation of **complicated** chlamydia diagnoses into age groups. Adjustment number: 1	Assumes that the age distribution of diagnoses for complicated chlamydia in 2000 to 2008 was equivalent to that seen for uncomplicated chlamydia during the same period.	1) A Kolmogorov-Smirnov test was used to statistically compare the uncomplicated and complicated chlamydia diagnosis distributions. This test showed no significant difference between distributions. 2) Alternatively, we could have reallocated complicated diagnoses captured between 2000 and 2008 according to the distribution of complicated diagnoses found in 2009. However, the results of a sensitivity analysis showed limited difference between methods (maximum percentage difference of 0.3% (range of 0.04–0.3%)).
Reallocation of **tests** between 2003 and 2008 according to the age group distribution in 2009. Adjustment number: 1	Assumes that the age distribution of tests between 2003 and 2008 was equivalent to that seen in 2009.	The rationale for this is based on two other observations: 1) The age distribution for chlamydia diagnoses coming from specialist services between 2003 and 2008 were comparable to the age distribution of diagnoses in 2009 (ANOVA test non-significant). 2) There was no variation in the age distribution for chlamydia tests coming from specialist services between 2009 and 2012 (ANOVA test non-significant).
Imputing data for 15 to 24-year-olds before 2008 and for > 24–year-olds before 2012 in **non-specialist** services. Adjustment number: 2	Assumes that all testing in non-specialist services for 15–24-year-olds before 2008, and in > 24-year-olds before 2012 followed a similar trend to that found in GP services.	We considered it a reasonable assumption that any changes seen in GP settings would also be reflected in other non-specialist services. Alternatively, we could have based this on the trend seen in specialist services. However, results from an audit of waiting times in specialist services show large increases in access to specialist services over this period following the first National Sexual Health Strategy [18]. Increases in testing outside of specialist services are therefore unlikely to have been of the same magnitude.
Imputing the number of chlamydia **tests** in **specialist** services between 2000 and 2002. Adjustment number: 2	Assumes a consistent trend in positivity over time from 2000 to 2008.	This adjustment was based patterns seen within later years of the data. The trend in positivity on which this adjustment was made was calculated using 2003–2008 data, rather than 2003–2012, being the period before full implementation of the NCSP and the GUMCAD surveillance system, which may have led to some changes in the available data.
Allowing for **referrals** from non-specialist to specialist services. Adjustment number: 3	Assumes a constant rate of referrals between non-specialist to specialist services between 2000 and 2012.	Our assumption is consistent with a previous study [21], which reported a steady referral rate between 2000 and 2004 from GP settings into specialist services.

ANOVA: analysis of variance; GP: general practice; GUMCAD:  Genitourinary Medicine Clinic Activity Dataset; NCSP: National Chlamydia Screening Programme.

^a^Adjustment number refers to the numbers found in the flowchart in Figure 2.

### Unknown age group or sex

Due to missing fields or aggregated reporting, tests and diagnoses could be reported without known sex or age group, therefore in these instances tests and diagnoses were reallocated according to the age and sex distributions seen in each year (see adjustment number 1 in Figure 2 and Table 1).

Between 2000 and 2008, diagnoses coming from specialist services were coded as either ‘uncomplicated’ or ‘complicated’ chlamydia (i.e. complicated when diagnosed with chlamydial PID and epididymitis). Complicated chlamydia diagnoses were not reported by age group. Based on a two-sample Kolmogorov-Smirnov test, the age distributions for ‘complicated’ and ‘uncomplicated’ chlamydia diagnoses in specialist services between 2009 and 2011 were not significantly different (borderline, p = 0.053). We therefore assumed the distributions were not significantly different between 2000 and 2008 and the ‘complicated’ diagnoses were reallocated into age groups according to the age distribution of ‘uncomplicated’ diagnoses.

Between 2003 and 2008, chlamydia tests in specialist services were reported by sex but not by age group. We therefore reallocated tests reported during this period into age groups according to the age group distribution of tests in 2009. This is based on analysis of variance tests (ANOVA) showing a non-significant difference between the age distribution of diagnoses between 2003 and 2012 (p = 1.0) and a non-significant difference between the age distribution of tests between 2009 and 2012 (the years where tests by age were reported, p = 0.9).

### Non-reported data: age group

During the analysis period, there are two major gaps in reporting where no data were collected through national monitoring and surveillance systems: (i) Before 2003, the number of chlamydia tests in specialist services were not collected; and (ii) in non-specialist services, data on chlamydia tests and diagnoses were incomplete before 2012, this included non-reported data for 15–24-year-olds before 2008 and for > 24-year-olds before 2012. To produce plausible estimates of total activity during these periods we imputed these data (see adjustment number 2).

Firstly, in order to impute the number of chlamydia tests in specialist services between 2000 and 2002, we used logistic regression to estimate the linear trend in positivity (percentage of chlamydia tests resulting in a positive diagnosis) between 2003 and 2008. Using the trend in positivity observed between 2003 and 2008, we predicted the positivity for 2000 to 2002. The model-predicted positivity trends were applied to the estimated diagnoses in order to estimate the numbers of tests in each year and age group from 2000 to 2002.

Secondly, we constructed minimum and maximum estimates of chlamydia testing coverage and diagnosis rates carried out in non-specialist services for 2000 to 2011 to allow for the uncertainty arising from non-reported data. Minimum estimates of chlamydia testing coverage and diagnosis rates were based on data available in the datasets (NCSP statistical returns and aggregated laboratory returns), combined with test and diagnosis rates derived from GP settings (CPRD) in those years where data were incomplete. To estimate maximum activity in non-specialist services, we used Poisson regression to estimate trends in test and diagnosis rates in the period where data from non-specialist services were incomplete or not reported. We then applied these model-estimated incidence rate ratios to the most recent ‘complete’ year of non-specialist services data (2008 for 15–24-year-olds; 2012 for > 24-year-olds).

### Referrals from non-specialist to specialist services

Individuals cannot be followed between non-specialist and specialist services in the datasets as different identifiers are used. Since 2012, a diagnostic code to indicate referrals from non-specialist services with a chlamydia diagnosis into specialist services was introduced (C4X code) [18,19]. We calculated the proportion of referrals in 2012, which ranged from 3.8% to 15.6% by age group. In both the minimum and maximum estimates of activity in specialist services, testing coverage and diagnosis rates were adjusted to allow for potential duplication between services, based on the proportions of referrals in 2012, assuming the rate of referrals was constant across the period (see adjustment number 3). While it is feasible that this has changed, this was considered a reasonable assumption as Hughes et al. reported a steady referral rate of 10% in 2000 to 2004 from GPs into specialist services [20], which is similar to the overall referral rate calculated for 2012 (8.4%).

### Men who have sex with men

This dataset was compiled with an aim to mathematically model heterosexual transmission of chlamydia. MSM were therefore removed from the minimum-activity estimate for specialist services (see adjustment number 4). Sexual orientation is not available for tests and diagnoses outside of specialist services, so this could not be adjusted for.

## Results

**Figure 3:**
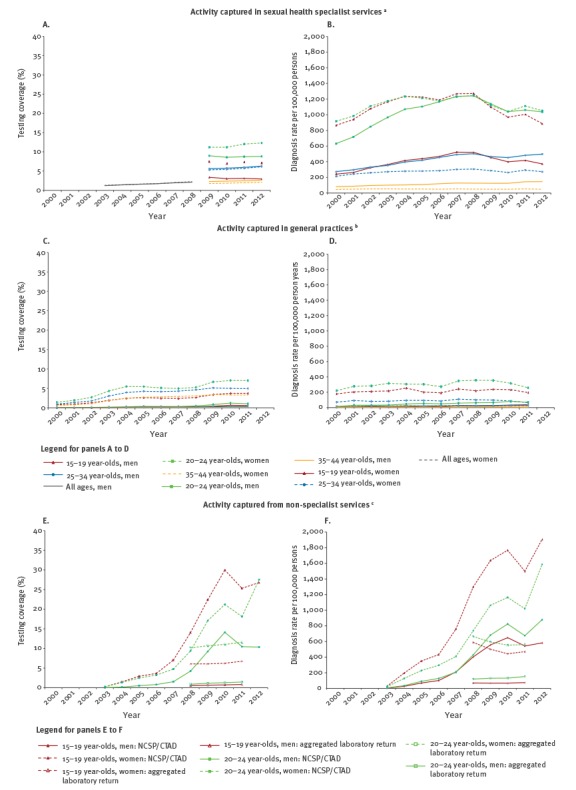
Reported rates of chlamydia tests and diagnoses captured in specialist sexual health services and non-specialist services by sex and age group, Panels A), C) and E) show tests; Panels B), D) and F) show diagnosis rates, England, 2000–2012

CPRD: Clinical Practice Research Datalink; CTAT: Chlamydia Testing Activity Dataset; GUMCAD: Genitourinary Medicine Clinic Activity Dataset; NCSP: National Chlamydia Screening Programme.

^a^ All ages for tests done in specialist services include 15- to 90-year-olds; testing activity was not broken down by age pre-2009. Diagnoses captured in specialist services incorporate both uncomplicated and complicated chlamydia diagnoses. Testing data in specialist services are available from 2003 to 2012 and diagnosis data are available from 2000 to 2012.Data captured in specialist services are from the English monitoring and surveillance systems: KC60 statistical return (2000 to 2008) and GUMCAD (2009 to 2012).

^b^ Activity rates within general practices are by person-years; data captured from 2000 to 2011 are for 15 to 44-year-olds. Data for general practices are captured in CPRD (2000 to 2011).

^c^ Testing and diagnosis data in non-specialist services capture data for 15 to 24-year-olds only. Data captured in non-specialist services are from English monitoring and surveillance systems: NCSP statistical return (2003 to 2011), aggregated laboratory return (2008 to 2011) and CTAD (2012). CTAD captures data for all ages, only data for 15 to 24-year-olds are displayed here.

Figure 3 shows the chlamydia testing and diagnosis rates by services according to the years and age groups before adjustments were made to the data.

Table 2 shows the compiled data sources for all genital chlamydia testing and diagnosis activity by age group and sex.

**Table 2:** Minimum and maximum estimates for chlamydia testing coverage and diagnosis rates in 15 to 44-year-old women and men across all service types.

		**Chlamydia testing coverage in women (per 100 persons)**								**Chlamydia testing coverage in men (per 100 persons)**							
		** Minimum estimates**				** Maximum estimates**				** Minimum estimates**				** Maximum estimates**			
		**15–19-year‑olds**	**20–24-year‑olds**	**25–34-year‑olds**	**35–44-year‑olds**	**15–19-year‑olds**	**20–24-year‑olds**	**25–34-year‑olds**	**35–44-year‑olds**	**15–19-year‑olds**	**20–24-year‑olds**	**25–34-year‑olds**	**35–44-year‑olds**	**15–19-year‑olds**	**20–24-year‑olds**	**25–34-year‑olds**	**35–44-year‑olds**
**Year**	**2000**	3.1	5.5	2.7	1.1	9.7	11.1	5.4	2.4	1.1	2.9	2.0	0.8	2.0	3.9	2.5	1.0
	**2001**	3.8	6.6	3.6	1.5	11.1	12.8	6.2	2.7	1.3	3.5	2.2	0.8	2.4	4.7	2.8	1.2
	**2002**	4.8	8.5	4.3	1.9	13.0	14.9	7.0	3.1	1.6	4.4	2.6	1.0	3.1	5.8	3.3	1.4
	**2003**	6.3	11.2	6.0	2.8	14.9	17.1	7.9	3.5	2.0	5.4	3.0	1.1	3.8	7.0	3.6	1.5
	**2004**	8.5	14.1	7.3	3.6	16.8	19.3	8.9	3.9	2.4	6.2	3.5	1.3	4.7	8.1	4.3	1.8
	**2005**	10.3	15.6	8.0	4.0	19.0	21.5	9.9	4.4	3.1	7.1	4.0	1.5	5.6	9.2	4.8	2.0
	**2006**	11.1	16.3	8.2	4.1	21.2	23.8	10.9	4.9	3.5	7.8	4.3	1.6	6.8	10.4	5.4	2.2
	**2007**	15.1	18.7	9.0	4.5	24.2	27.2	12.4	5.5	5.5	9.3	4.9	1.8	8.4	12.1	6.2	2.5
	**2008**	27.3	30.4	9.9	4.8	27.3	30.4	13.8	6.2	10.2	13.4	5.3	2.0	10.4	13.9	6.8	2.8
	**2009**	35.7	38.8	10.4	5.2	35.7	38.8	14.8	6.8	16.1	18.8	5.3	2.0	16.3	19.3	7.1	2.9
	**2010**	43.0	43.3	10.4	5.2	43.0	43.3	16.0	7.5	23.9	23.3	5.4	2.1	24.0	23.9	7.5	3.1
	**2011**	39.3	41.6	10.6	5.3	39.3	41.6	17.5	8.4	19.0	19.8	5.4	2.1	19.2	20.6	8.1	3.4
	**2012**	33.9	39.9	19.2	9.3	33.9	39.9	19.2	9.3	14.2	18.2	7.7	3.0	14.5	19.2	8.8	3.7
		**Chlamydia diagnosis rates in women (per 100,000 persons)**								**Chlamydia diagnosis rates in men (per 100,000 persons)**							
		**Minimum estimates**				**Maximum estimates**				**Minimum estimates**				**Maximum estimates**			
		**15–19-year-olds**	**20–24-year-olds**	**25–34-year-olds**	**35–44-year-olds**	**15–19-year-olds**	**20–24-year-olds**	**25–34-year-olds**	**35–44-year-olds**	**15–19-year-olds**	**20–24-year-olds**	**25–34-year-olds**	**35–44-year-olds**	**15–19-year-olds**	**20–24-year-olds**	**25–34-year-olds**	**35–44-year-olds**
**Year**	**2000**	891.1	1,043.1	256.7	56.5	2,488.6	1,909.5	502.6	113.3	235.7	621.8	268.1	78.8	387.1	815.7	343.4	113.5
	**2001**	987.5	1,162.3	305.5	62.9	2,589.5	2,012.8	530.8	117.2	255.7	717.7	291.7	81.8	431.7	925.5	372.9	120.8
	**2002**	1,130.7	1,293.3	311.3	67.0	2,756.4	2,182.7	548.9	120.2	318.6	849.1	330.6	87.8	519.3	1,088.3	412.1	129.4
	**2003**	1,244.1	1,402.0	327.8	68.3	2,871.2	2,288.0	564.6	120.0	356.3	969.4	345.8	94.0	589.8	1,241.3	440.2	134.6
	**2004**	1,508.3	1,538.9	344.7	73.4	2,952.0	2,388.4	572.3	118.4	424.5	1,108.8	390.5	94.8	674.9	1,383.8	490.9	138.5
	**2005**	1,576.5	1,588.3	344.6	64.6	2,968.4	2,408.1	574.8	118.9	489.4	1,189.2	408.4	97.2	736.8	1,460.3	521.0	143.7
	**2006**	1,594.2	1,568.0	340.3	68.7	2,954.7	2,417.4	581.6	115.0	535.0	1,267.3	439.2	103.8	811.2	1,572.9	565.8	155.2
	**2007**	2,022.5	1,782.3	381.3	74.7	3,034.0	2,510.8	597.1	119.4	704.0	1,415.6	474.6	110.7	912.5	1,696.3	611.7	164.2
	**2008**	2,979.6	2,516.9	379.3	68.4	2,979.6	2,516.9	604.0	117.3	946.9	1,716.1	477.6	105.4	957.6	1,751.5	632.6	163.7
	**2009**	3,033.9	2,621.4	355.2	59.8	3,033.9	2,621.4	587.2	116.4	1,020.2	1,854.3	435.3	99.8	1,032.6	1,895.4	605.8	163.5
	**2010**	2,972.8	2,598.7	318.9	61.5	2,972.8	2,598.7	566.2	115.8	1,049.4	1,879.1	416.3	94.5	1,063.8	1,928.0	606.0	163.2
	**2011**	2,786.6	2,547.1	327.2	64.9	2,786.6	2,547.1	599.3	120.0	970.9	1,754.0	414.8	91.4	989.9	1,829.5	647.0	184.1
	**2012**	2,791.8	2,639.5	587.6	117.3	2,791.8	2,639.5	587.6	117.3	935.5	1,830.2	589.7	130.0	953.8	1,913.7	678.7	186.8

Between 2000 and 2008, there was a large range between minimum and maximum estimate scenarios for both testing coverage and diagnosis rates. For example, in 15–19-year-old women in 2000, diagnosis rates ranged from 891 to 2,489 diagnoses per 100,000 persons. In both scenarios and across all age groups (15–44-year-olds), estimated testing coverage and diagnosis rates were higher in women than men.

In women and men of all age groups (15–44-year-olds), there was an overall increase in chlamydia testing coverage and diagnosis rates from 2000 to 2012 in all settings. The greatest increases in both testing coverage and diagnosis rates were seen among 15 to 24-year-olds, with the greatest increase in this age group found between 2008 and 2010. From 2010 there was a small decline in testing and diagnosis rates among 15–24-year-olds. Whereas the minimum estimate scenario showed a large increase in estimated diagnosis rates in women from 2000, a more gradual increase was seen for the maximum estimate scenario. In both minimum and maximum estimate scenarios, estimated diagnosis rates were relatively stable from 2008 to 2012 in women and men.

## Discussion

We used data captured by a range of monitoring and surveillance systems to construct a dataset representing all genital chlamydia testing and diagnosis activity taking place in England between 2000 and 2012. Gaps in the available data mean there is considerable uncertainty around the total amount of testing and diagnoses in the years before 2008. We therefore constructed minimum and maximum estimates to acknowledge this but set bounds on the uncertainty within the data.

The changes seen in chlamydia testing and diagnosis rates are in line with the evolution of the NCSP and chlamydia testing in England. An overall increase in testing and diagnosis rates were observed among 15–44-year-olds, which is likely due to increased awareness and better practice of chlamydia testing in England. The greatest increase in rates were observed in 15–24-year-olds, relating to an increase in opportunistic testing targeted in under-25-year-olds, as part of the NCSP from 2003. A sharp increase was seen from 2008 due to the nationwide implementation of the NCSP in 2008, accompanied by national targets for testing coverage. The decline in testing rates from 2010 may be explained, in part, by the changes in targets for testing during this period [21].

The constructed dataset resulting from our work has several applications. From these findings we have a better understanding of the potential effects of the NCSP on testing coverage and diagnoses. However, this does not provide the complete picture, as further insight is needed to understand how prevalence and/or incidence have changed in the context of the programme. Mathematical modelling offers a means to do this and our constructed data can be used to parameterise such models to better quantify the public health impact of the NCSP. Our data can also be used to parameterise and validate mathematical models designed to explore optimum approaches to chlamydia control (e.g. by varying rates of partner notification or changing the population tested). The findings of such modelling would be of benefit beyond England as the principles of chlamydia epidemiology and likely impact of different chlamydia control measures would likely hold across many different countries. Findings from such analyses could therefore inform chlamydia control activities in Europe and elsewhere.

In addition, these data can serve as a reference for interpreting trends in chlamydia-related complications. For example, trend in rates in PID, a complication associated with STIs including chlamydia, can be compared with chlamydia rates and determine if any changes reflected in one may be reflected in the other [22]. This is important for evaluation of the NCSP as an aim of the programme is to reduce associated complications through opportunistic screening. Again, findings from such studies would have relevance beyond England, as a better understanding the impact of chlamydia control on complications is needed to inform decisions about how best to approach chlamydia control [11].

The main strength of our analysis is the use of data from well-documented and established datasets, in which the changes in coding, testing practices and gaps in the data are understood. There are, however, some limitations. While every effort has been made to use data-driven and evidence-based assumptions to adjust for missing data, it is possible that our estimates have resulted in some over- or under- estimation of activity. We used data on referral patterns in 2012 to de-duplicate testing episodes between settings. However de-duplication of testing or diagnosis episodes is likely to be incomplete. For example, if an individual visited two different specialist services for the same testing episode, it would not be possible to remove the duplicate record. For this analysis, only tests undertaken by publicly-funded services have been counted, as private tests are excluded as part of the data collection specification [13]. When dealing with data where the age and sex were unknown, we used statistical tests to guide our decision about the most appropriate distribution to apply to the data. In the case of complicated chlamydia diagnoses, our finding was of borderline significance meaning that we may have incorrectly allocated by age group. However, as complicated diagnoses made up a minority of diagnoses from specialist sexual health services over this period (< 3.5%) this is unlikely to have made a substantial difference to the resulting dataset. Sensitivity analysis showed that applying an alternative assumption (i.e. reallocate according to the age distribution seen in 2009) made negligible difference. It is feasible that reallocating tests of unknown age in 2003 to 2008 according to the age-group distribution of tests in 2009 may have introduced error, as the NCSP was being rolled out in these earlier years.

During the analysis period, more sensitive and specific chlamydia tests have become available [23]. There is potential for both false negative and false positive results to have occurred over this period due to imperfect sensitivity and specificity of enzyme immunoassay (EIA) tests in particular, which were phased out in England during the mid-2000s [23]. We did not adjust our estimated diagnosis rates for test performance, as the test platforms used were not routinely collected and the exact performance characteristics are difficult to apply given the absence of an agreed gold standard [24].

Given the nature of this work and the absence of data, there are limited sources in the literature to validate our estimates. However, findings from the second National Survey of Sexual Attitudes and Lifestyles (Natsal-2, a stratified probability survey of British general population carried out in 1999–2000) are consistent with the estimated diagnosis rates calculated in this work. For example among 20–24-year-old Natsal-2 participants who had ever had sex, 0.7% (95% confidence interval 0.2–2.0%) of men and 1.7% (1.0–2.9%) of women reported having been diagnosed with chlamydia in the last year [25]. In our constructed dataset, assuming that each diagnosis represents an individual, the minimum and maximum estimates of percentage tested in 2000 was 0.6% to 0.8% in 20–24-year-old men and 1.0% to 1.9% in women, thus falling within the 95% confidence limits of the survey-based estimates. Currently, we do not have other external validation methods.

While there is uncertainty in the absolute numbers of chlamydia tests and diagnoses estimated in the earlier years of our analysis period, it is highly likely that testing and diagnosis rates did increase from at least the early 2000s onwards. This is especially the case among under-25-year-olds as the target age group for the NCSP, which was implemented on a phased basis in 2003 and achieved national implementation by 2008 [9,26]. Data from the second and third Natsal studies in 1999–2000 (Natsal-2) and 2009–2011 (Natsal-3)) indicate that diagnoses have increased substantially over the decade, with the percentage of 16–24-year-olds who reported a chlamydia diagnosis in the last 5 years increasing from 1.5% (1.2–1.8) to 4.1% (3.6–4.7) in women and from 0.8% (0.5–1.1) to 4.0% (3.4–4.8) in men [4]. It is likely therefore that our maximum scenario estimates of diagnoses in earlier years in women are an overestimate. However, we retained this liberal estimate of diagnoses in the maximum scenario as we could not narrow these plausible ranges further on the basis of the available data.

The problem of missing data from chlamydia surveillance systems is not one limited to England. Surveillance systems across Europe are known to vary in their completeness with respect to diagnoses, and few countries routinely collect and report data on testing, which is invaluable in interpreting trends in diagnoses of chlamydia, given that it is a largely asymptomatic infection. So, could our approach be applied to other settings? Our analysis has highlighted the multiple complexities in undertaking such an exercise, even in the context of England, where surveillance systems are more complete than many others in Europe and have included testing denominators for several years [8]. However, it is possible that multiple data sources from other countries may be combined in a similar fashion to ascertain minimum and maximum estimates, through application of reasonable assumptions about the completeness of the data or relationships between them. Such an undertaking would need to be carried out on a case by case basis, involving in-country experts with in-depth knowledge of data collection systems as well as an understanding of healthcare systems and changes in policy and practice over time.

## Conclusions

Our analysis provides plausible comprehensive estimates of chlamydia testing and diagnosis activity in England from 2000. Since 2012, developments in monitoring and surveillance systems for chlamydia and other STI in England, embodied by CTAD and GUMCADv2, have allowed a comprehensive record of chlamydia testing and diagnosis activity from a single data source with far less uncertainty, enabling more robust assessment and evaluation of the English NCSP in future years. It is possible that similar methods to ours could be used for data captured in surveillance systems in applicable countries across Europe, however, our analysis highlights the potential complexities faced when estimating testing and diagnosis activity from multiple and changing data sources. When examining trends over time using monitoring and surveillance data or compiling data from different sources, we recommend that known limitations be carefully considered and addressed where possible.
